# Dietary supplemental vitamin B6 increases carnosine and anserine concentrations in the heart of rats

**DOI:** 10.1186/s40064-015-1074-8

**Published:** 2015-06-19

**Authors:** Sofya Suidasari, Tomoko Hasegawa, Noriyuki Yanaka, Norihisa Kato

**Affiliations:** Graduate School of Biosphere Science, Hiroshima University, Higashi-Hiroshima, 739-8528 Japan

**Keywords:** Vitamin B6, Carnosine, Anserine, Heart, UPLC–MS/MS

## Abstract

This study was performed to examine the effect of dietary level of vitamin B6 on the concentrations of carnosine and anserine, antioxidants, in the heart of rats. Analysis using UPLC–MS/MS showed that the concentrations of these dipeptides in the 7 and 35 mg pyridoxine HCl/kg groups were significantly higher than those in the 1 mg pyridoxine HCl/kg group, implying the novel role of dietary vitamin B6 as a determinant of the dipeptides favorable for heart.

## Background

Increasing evidence indicates that lower levels of vitamin B6 (B6) are related to the risk of coronary heart disease and atherosclerosis (Robinson et al. 1998), with the anti-inflammatory effect of B6 considered to be at least partially responsible for its protective effect against these diseases (Friso et al. 2004). Furthermore, B6 exerts an anti-ischemic effect in the heart by blocking purinergic receptors (Dhalla et al. 2013). However, the effect of B6 on the heart per se remains obscure.

Pyridoxal 5′-phosphate (PLP), the active form of B6, acts as a co-factor for several enzymes involved in amino acid metabolism. In this study, we hypothesized that dietary supplemental B6 affects heart dysfunction by modulating amino acid metabolism. Accordingly, we investigated the effects of low to high B6 diets on the concentrations of free amino acids and related metabolites in the heart of rats. Here we provide the first evidence that dietary supplemental B6 to a low B6 diet markedly elevates heart concentrations of carnosine and anserine, putative antioxidants favorable for heart (Boldyrev et al. 2013).

## Methods

Male Sprague–Dawley rats (4 weeks old, Charles River Japan, Hino, Japan) were maintained in accordance with the Guide for the Care and Use of Laboratory Animals established by Hiroshima University. The rats were housed in metal cages in a temperature-controlled room (24 ± 1°C) under a 12-h light/dark cycle (lights on, 0800–2000 hours). The rats had free access to food and deionized water. The basal diet was described previously (Masisi et al. 2012). Pyridoxine (PN) HCl was supplemented to the basal diet at concentrations of 1, 7, or 35 mg/kg diet. The 7 mg/kg diet is the recommended level of dietary B6 (Reeves et al. 1993). Meanwhile, 1 mg PN HCl/kg diet is reported to be the minimum level required for preventing growth depression caused by B6 deficiency (Coburn 1994). After being fed a commercial non-purified diet (MF, Oriental Yeast, Tokyo, Japan) for 1 week, 24 rats (average body weight 70 g) were randomly divided into three groups receiving 1, 7, or 35 mg PN HCl/kg diet (*n* = 8/group) for 6 weeks. The animals were killed by decapitation under diethyl ether anesthesia. Serum was collected from truck blood and stored at −60°C. Hearts were quickly dissected, frozen in liquid nitrogen, and immediately stored at −80°C.

For analysis of carnosine and anserine in heart by ultra-performance liquid chromatography coupled with tandem mass spectrometry (UPLC–MS/MS), the frozen hearts were homogenized with cold (4°C) 3% (w/v) sulfosalicylic acid to precipitate protein. After centrifugation at 1,000×*g* at 4°C for 30 min, the supernatants were collected and filtered through a 0.22-µm-pore membrane filter and immediately stored at −80°C until analysis. The supernatant samples of each two rats from the same group of eight rats were combined to obtain the pooled four samples for the analysis. Separation was performed on an UPLC–MS/MS system as described previously (Waterval et al. 2009), with a slight modification. Analysis of PLP concentrations in the serum and heart was performed as described previously (Masisi et al. 2012). Serum samples were analyzed using a commercial carnosine ELISA kit (USCN Life Science, Inc., Wuhan, China). Data are expressed as mean ± SE. Tukey’s multiple-range test was used to compare means after one-way ANOVA. Statistical significance of the difference between means was estimated at *P* < 0.05.

## Results and discussion

**Figure 1:**
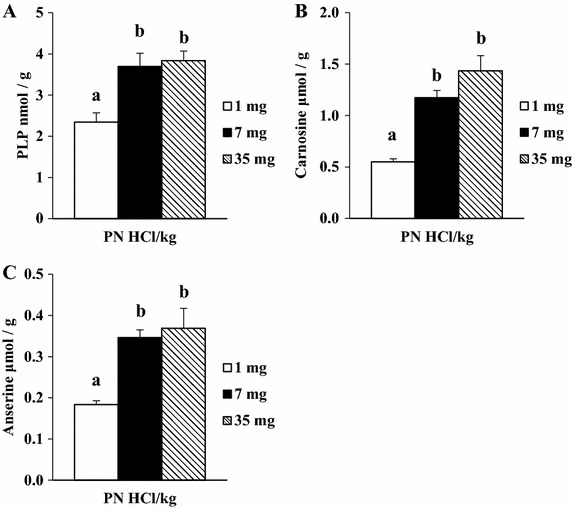
Effect of dietary levels of B6 (PN HCl) on concentrations of PLP (**A**), carnosine (**B**), and anserine (**C**) in the heart of rats. Mean ± SE (*n* = 5 for PLP; *n* = 4 for carnosine and anserine). For the analysis of carnosine and anserine, the supernatant samples of each two rats from the same group of eight rats were combined to obtain the pooled four samples. Values with *different superscript* are significantly different by Tukey’s multiple-range test (*P* < 0.05).

**Figure 2:**
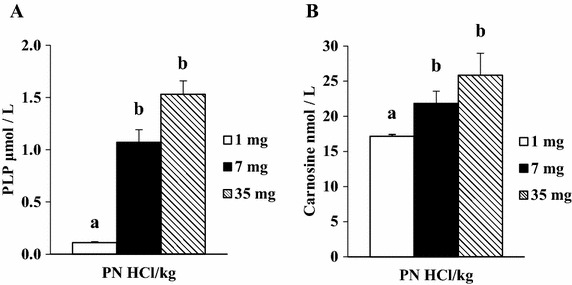
Effect of dietary levels of B6 (PN HCl) on concentrations of PLP (**A**) and carnosine (**B**) in the serum of rats. Mean ± SE (*n* = 5 for PLP; *n* = 8 for carnosine). Values with *different superscript* are significantly different by Tukey’s multiple-range test (*P* < 0.05).

Dietary manipulation did not affect food intake (1, 7, and 35 mg PN HCl/kg groups: 731 ± 10, 787 ± 15, and 772 ± 24 g/6 weeks, respectively, *P* > 0.05) or final body weight (412 ± 9, 444 ± 9, and 437 ± 13 g, respectively, *P* > 0.05). Heart weight in the 1, 7, and 35 mg PN HCl/kg groups were 1.14 ± 0.05, 1.20 ± 0.03, and 1.16 ± 0.03 g, respectively, *P* > 0.05). PLP concentrations in the heart and serum in the 7 and 35 mg PN HCl/kg groups were higher (*P* < 0.01) than those in the 1 mg PN HCl/kg group (Figures 1A, 2A, respectively). There was no significant difference in the PLP concentrations between the 7 and 35 mg PN HCl/kg groups.

Our preliminary study using the amino acid analyzer showed marked alterations in the concentrations of carnosine and anserine in the heart depending on the level of dietary B6. Thus, further analysis using UPLC–MS/MS was conducted to determine the concentrations of these dipeptides.

The 7 and 35 mg PN HCl/kg groups had markedly higher concentrations of carnosine (+114 and +162%, respectively, *P* < 0.01, Figure 1B) and anserine (+89 and +101%, respectively, *P* < 0.01, Figure 1C) than those in the 1 mg PN HCl/kg group. The concentrations of these peptides in the 7 and 35 mg PN HCl/kg groups did not differ. Carnosine is reported to exert anti-oxidant, anti-inflammatory, and anti-ischemic effects on the heart (Boldyrev et al. 2013; Fleisher-Berkovich et al. 2009; Stvolinsky and Dobrota 2000). It has biochemical capacities such as pH-buffering and metal ion chelation (Boldyrev et al. 2013). In cardiac myocytes, carnosine is suggested to be a modulator of intracellular calcium and contractility (Roberts and Zaloga 2000; Zaloga et al. 1997). Anserine also exerts anti-oxidant and anti-inflammatory effects (Song et al. 2014). Thus, the present results imply that dietary supplemental B6, correcting low B6 status, might be favorable for muscle function by elevating the histidyl-dipeptides.

B6 supplementation resulted in higher serum concentration of carnosine, with a significant difference between the 35 mg PN HCl/kg group and the 1 mg PN HCl/kg group (*P* < 0.05, Figure 2B). It has been suggested that higher concentrations of carnosine on account of exercise decrease blood pressure in rats and humans (Nagai et al. 2012). A recent study suggested that plasma carnosine is involved in preventing early-stage lipid oxidation in circulation (Stegen et al. 2015). Thus, elevated serum carnosine following B6 supplementation may partially relate to the favorable effect of B6 on circulation. Nevertheless, further study is required to examine this possibility.

## Conclusion

In conclusion, the present study indicated that inadequate level of B6 in the diet (1 mg PN HCl/kg) causes lower concentrations of heart carnosine and anserine, putative protective factors against heart dysfunction, compared to the recommended level of B6 and high level of B6 (7 and 35 mg PN HCl/kg, respectively). Thus, adequate intake of B6 is likely to be necessary for maintaining the peptides in the heart. This finding is of importance because recent evidence suggests marginal B6 deficiency is common in the USA and Japan (Morris et al. 2008; Murakami et al. 2008). Further study is required to determine the underlying mechanisms by which B6 supplementation alters these dipeptides and thus exerts heart-protective effects.

## Abbreviations

B6: vitamin B6
PLP: pyridoxal 5′-phosphate
PN: pyridoxine
UPLC–MS/MS: ultra-performance liquid chromatography coupled with tandem mass spectrometry
